# EPR-Effect Enhancers Strongly Potentiate Tumor-Targeted Delivery of Nanomedicines to Advanced Cancers: Further Extension to Enhancement of the Therapeutic Effect

**DOI:** 10.3390/jpm11060487

**Published:** 2021-05-28

**Authors:** Waliul Islam, Shintaro Kimura, Rayhanul Islam, Ayaka Harada, Katsuhiko Ono, Jun Fang, Takuro Niidome, Tomohiro Sawa, Hiroshi Maeda

**Affiliations:** 1 Department of Microbiology, Graduate School of Medical Sciences, Kumamoto University, Kumamoto 860-8556, Japan; bcmb.waliul@gmail.com (W.I.); s.kimura@stateart.co.jp (S.K.); onokat@kumamoto-u.ac.jp (K.O.); sawat@kumamoto-u.ac.jp (T.S.); maedabdr@sweet.ocn.ne.jp (H.M.); 2 BioDynamics Research Foundation, Kumamoto 862-0954, Japan; 3 StateArt Inc., Tokyo 103-0012, Japan; 4 Faculty of Pharmaceutical Sciences, Sojo University, Kumamoto 860-0082, Japan; rayhanulislam88@gmail.com; 5 Faculty of Advanced Science and Technology, Kumamoto University, Kumamoto 860-8555, Japan; 144t1817@gmail.com (A.H.); niidome@kumamoto-u.ac.jp (T.N.); 6 Tohoku University, Sendai 980-8572, Japan

**Keywords:** isosorbide dinitrate, sildenafil citrate, EPR effect, EPR-effect enhancers, heterogeneity of the EPR effect, nitric oxide donors, tumor blood flow

## Abstract

For more than three decades, enhanced permeability and retention (EPR)-effect-based nanomedicines have received considerable attention for tumor-selective treatment of solid tumors. However, treatment of advanced cancers remains a huge challenge in clinical situations because of occluded or embolized tumor blood vessels, which lead to so-called heterogeneity of the EPR effect. We previously developed a method to restore impaired blood flow in blood vessels by using nitric oxide donors and other agents called EPR-effect enhancers. Here, we show that two novel EPR-effect enhancers—isosorbide dinitrate (ISDN, Nitrol^®^) and sildenafil citrate—strongly potentiated delivery of three macromolecular drugs to tumors: a complex of poly(styrene-co-maleic acid) (SMA) and cisplatin, named Smaplatin^®^ (chemotherapy); poly(N-(2-hydroxypropyl)methacrylamide) polymer-conjugated zinc protoporphyrin (photodynamic therapy and imaging); and SMA glucosamine-conjugated boric acid complex (boron neutron capture therapy). We tested these nanodrugs in mice with advanced C26 tumors. When these nanomedicines were administered together with ISDN or sildenafil, tumor delivery and thus positive therapeutic results increased two- to four-fold in tumors with diameters of 15 mm or more. These results confirmed the rationale for using EPR-effect enhancers to restore tumor blood flow. In conclusion, all EPR-effect enhancers tested showed great potential for application in cancer therapy.

## 1. Introduction

The enhanced permeability and retention (EPR) effect is believed to be a universal mechanism occurring in most solid tumors and a key issue for selective delivery of nanomedicines to tumors [1,2,3,4,5,6]. Suppressed blood flow or obstructed blood vessels in advanced cancers lead to heterogeneity of the EPR effect [7,8,9,10,11,12]. Criticisms of the EPR effect were recently raised [13,14], probably because of inaccurate understanding of the EPR effect together with the use of inappropriate nanomedicines, particularly those lacking good stability in vivo and those with an inadequate or poor experimental design [7,8,9]. For instance, if the release of active pharmaceutical ingredients from liposomes is too slow because the complexes are very stable, even though they accumulates in tumors by EPR effect, the therapeutic outcome is poor [7,8,9,10,11,12,15].

The EPR effect was first demonstrated in mouse tumor models in which the tumor size was usually smaller than 10 mm and the tumors were highly vasculated; nanomedicines thus had high permeability. In contrast, human tumors diagnosed in clinical situations are frequently larger than 3 mm and up to 10 cm or more. In such large tumors, blood flow is often suppressed or blood vessels are occluded because of the formation of vascular clots or thrombi [9,10,11,12,15,16,17,18]. This blood-flow suppression thus results in little or no drug delivery and, therefore, a highly limited EPR effect [9,12,15,16,17,18]. However, Ding et al. observed that more than 87% of human renal tumors manifested a considerable EPR effect with significant diversity and heterogeneity in different patients [19]. Also, Lee et al. reported that nanoparticles conjugated with positron-emitting radionuclei such as ^64^Cu resulted the EPR effect in breast cancer, including metastatic cancer [20]. We demonstrated similar results by using arterial angiography of the polymer-conjugate drug SMANCS, i.e., neocarzinostatin (NCS) conjugated to poly(styrene-co-maleic acid) (SMA), in lipiodol [21,22,23,24]. In these situations, restoration of tumor blood flow led to successful treatments with nanomedicines [1,23,24]. The review article by Maeda covers these issues [24].

In our studies to overcome the problem of occluded blood flow in advanced tumors, we achieved a breakthrough by using the nitric oxide (NO) donors nitroglycerin (NG), L-arginine (L-Arg), hydroxyurea (HU), and an ACE (angiotensin-converting enzyme) inhibitor as well as other agents including carbon monoxide (CO)-releasing micelles, such as SMA-encapsulated CO-releasing molecule-2 (SMA/CORM2) and polyethylene glycol-hemin (PEG-hemin) [7,8,9,10,11,12,25,26,27]. Some of these NO-releasing agents are routinely used in clinical situations. They generate NO in tumors in a selective manner so that tumor blood vessels open mostly through the effect of vasodilation. NO thereby facilitates the EPR effect and delivery of drugs to tumors [7,9,10,11,12,25]. These enhancers increased drug delivery to different implanted tumors (S180, C26, and B16) two- to three-fold in mice. They also improved the therapeutic effect two- to three-fold in autochthonous colon tumors induced chemically with azoxymethane and 2% dextran sodium sulfate in mice and 7,12-dimethylbenz[*a*]anthracene-induced advanced breast tumors in rats [9,12,25]. These two tumor models are similar to naturally occurring tumors and tumors seen in clinical conditions. 

In this study, we investigated three EPR-effect enhancers—isosorbide dinitrate (ISDN), sildenafil citrate, and L-Arg—in C26 tumor models in mice, which exhibit less tumor blood flow and are not easy to cure compared to S180 tumor model. ISDN is an organic nitrate compound used to treat angina pectoris, heart failure, and esophageal spasms; to treat and prevent cardiac infarction; and to restore blood flow to the heart [28,29,30]. ISDN is absorbed at several sites, including the gastrointestinal tract, mucous membranes, and skin, depending on the formulation [31], after which the nitroxyl (-O-NO_2_) moiety releases the nitrite ion [32]. Nitrite (NO_2_) is converted to NO by nitrite reductase [32,33]. A patient with lung adenocarcinoma with multiple tumor masses was treated with Nitrol^®^ (ISDN) by means of arterial infusion of SMANCS/lipiodol (0.5 mg/0.5 mL total), the outcome being marked tumor suppression even after only one infusion of SMANCS/lipiodol. This patient remained in good health and free of tumors, as judged by computed tomography (CT), after at least one year and six months [11]. However, a positive effect of the intravenous infusion of Nitrol^®^ and polymer-drug conjugate in an aqueous formulation was not fully demonstrated in this clinical setting. In contrast, sildenafil citrate, another EPR enhancer recently described to have a positive effect in tumor delivery [34], is widely used for male erectile dysfunction. It is a selective inhibitor of phosphodiesterase type 5 that enhances extravasation in target tissues by inhibiting cGMP degradation [35], with results similar to those of NO; however, it does not contain a nitro group.

We report here our utilization of these vascular mediators in combination with three macromolecular drugs to increase delivery of the drugs to tumors and thereby improve therapeutic efficacy in advanced C26 tumors in mice. These three drugs tested are the following: a complex of SMA encapsulated cisplatin (registered name Smaplatin^®^), in which cisplatin is used in cancer chemotherapy [36]; SMA glucosamine-conjugated boric acid complex (SGB-complex) that was designed for boron neutron capture therapy (BNCT) [37]; and poly(N-(2-hydroxypropyl methacrylamide) (P-HPMA) copolymer-conjugated zinc protoporphyrin (PZP), used for photodynamic therapy (PDT) [38]. Our data showed about two- to four-fold enhancement of therapeutic outcome for all these drugs. These findings again indicate the importance of EPR-effect enhancers to restore tumor blood flow for successful treatment of solid tumors.

## 2. Materials and Methods

### 2.1. Chemicals

ISDN was purchased from Eisai Co. Ltd., Tokyo, Japan. Sildenafil citrate was purchased from Yoshindo Co. Ltd., Toyama, Japan. *cis*-Diamminedichloroplatinum(II) (CDDP, cisplatin^®^) was purchased from Sigma-Aldrich, Tokyo, Japan. SMA (molecular size 5500–6500 Da) was obtained from KJ Chemicals, Tokyo, Japan. Smaplatin^®^, with a particle size of 100.2 nm as described previously [36]; SGB-complex, with a diameter of 12–15 nm, containing about 16–18% (*w*/*w*) glucosamine; and 7–8% (*w*/*w*) boric acid was synthesized by Maeda’s group [37]. P-HPMA-conjugated zinc protoporphyrin (PZP) [38] was developed previously for PDT. All other reagents and solvents of reagent grade were from commercial sources and were used without additional purification.

### 2.2. Animals, Cells, and Tumor Models

All animals used in in vivo studies were maintained at 22 ± 1 °C and 55 ± 5% relative humidity with a 12-h light/dark cycle. Each cage contained 4 mice in this study. All experiments were approved by the Animal Ethics Committee of Kumamoto University and carried out according to the Laboratory Protocol of Animal Handling, Kumamoto University, Kumamoto, Japan. Mice were randomly assigned to study groups, and endpoints of experiments were governed by tumor volume (up to ~4000 mm^3^). Male BALB/c mice, all 6 weeks old, were purchased from SLC, Shizuoka, Japan. 

For solid tumor model experiments, mouse colon cancer C26 cells were maintained and cultured in vitro by using Dulbecco’s Modified Eagle’s Medium (Wako Pure Chemical Industry, Osaka, Japan) and supplemented with 10% fetal bovine serum (Biosera, Kansas City, MO, USA) and antibiotics (100 U/mL penicillin and 100 μg/mL streptomycin) (Nacalai Tesque, Kyoto, Japan) in 5% CO_2_/air at 37 °C. Cultured C26 cells were collected and suspended in physiological saline to a concentration of 2 × 10^7^ cells/mL. We implanted 0.1 mL of cell suspension in the dorsal skin of BALB/c mice to obtain C26 solid tumors.

### 2.3. Enhancement of Drug Delivery by Using ISDN and Sildenafil Citrate in Advanced C26 Tumors

For this study, we utilized BALB/c mice, 6 weeks old, that had relatively large-sized (diameter of >15 mm) or advanced tumors. Tumor diameters measured about 15 mm at 15–18 days after injection of C26 cells into the dorsal skin. Those mice bearing tumors without wound, collapse, and necrosis were included in this study. PZP (10 mg/kg) was then infused intravenously (iv) or was infused as part of the combination treatment with EPR-effect enhancers, which were injected after the PZP infusion: ISDN at 30 mg/kg intraperitoneally (ip) or sildenafil citrate at 30 mg/kg subcutaneously (sc). At 24 h after the PZP infusion, mice were killed, and blood samples were withdrawn; other tissues, including the heart, lung, liver, spleen, kidney, intestine, and tumor, were collected after perfusion with 20 mL of phosphate-buffered saline to remove blood from the tissues. Each tissue sample was added to 100 mg/mL dimethyl sulfoxide and homogenized very well. Samples were then centrifuged at 12,000× *g* for 30 min, and supernatants were collected. Finally, the amounts of PZP in the plasma and each tissue were measured in supernatants by means of fluorescence spectroscopy (excitation wavelength 422 nm, emission wavelength 590 nm).

### 2.4. Improvement in Drug Delivery to Tumors by ISDN as Evaluated by Inductively Coupled Plasma Mass Spectroscopy (ICP-MS)

We used two drugs, SGB-complex and Smaplatin^®^, to quantify the increased drug delivery induced by ISDN. When tumor diameters were 15–16 mm, we injected 15 mg/kg SGB-complex (boric acid equivalent) or 10 mg/kg Smaplatin^®^ (cisplatin equivalent) into mice via the tail vein. The EPR-effect enhancer ISDN, at 30 mg/kg, was administered ip immediately after the drug injection. After 24 h, mice were killed, and blood samples and other tissues were collected as described above. For elemental quantification by ICP-MS, specimens of about 100 mg of tumor and normal tissues, including the liver, spleen, kidney, intestine, heart, lung, and skin, were excised and placed into test tubes, followed by the addition of a 1:1 mixture of concentrated nitric acid and sulfuric acid (0.25 mL), and samples were digested at 80 °C for 2 h. Samples were then cooled, 10 mL of deionized water was added to each tube, followed by vortexing, and then samples (about 1 mL) were analyzed by using ICP-MS. The amounts (parts per billion, ppb) of ^10^B and platinum in each tissue were measured and compared.

### 2.5. Ex Vivo Imaging of PZP with ISDN and Sildenafil Citrate in Advanced Mouse C26 Tumors

When tumor diameters were about 18 mm, we infused 5 mg/kg PZP (ZnPP equivalent) iv. We administered the EPR enhancer ISDN or sildenafil citrate immediately after the PZP infusion. After 24 h, mice were killed, and tumor tissues were removed and subjected to fluorescence imaging by IVIS (IVIS XR; Caliper Life Sciences, Hopkinton, MA, USA). As a positive control, we used L-Arg at 50 mg/mouse in combination with PZP in similar experimental settings.

### 2.6. Augmentation of the Therapeutic Effects of Micellar Anticancer Agents Used in Combination with EPR-Effect Enhancers

To evaluate the therapeutic results of using two EPR-effect enhancers (ISDN and sildenafil citrate) with Smaplatin^®^ or SGB-complex, we administered Smaplatin^®^ iv at 6 mg/kg as the high dose or 3 mg/kg as the low dose to mice with C26 tumors when the tumors had diameters of 10–12 mm. For the combination therapy with the low Smaplatin^®^ dose, we added ISDN at 30 mg/kg ip or sildenafil citrate at 10 or 30 mg/kg sc. For the combination therapy with the SGB-complex, 10 mg/kg or 5 mg/kg (boric acid equivalent) was infused iv, and immediately after the infusion, the enhancers were injected. In a control experiment, we investigated another EPR-effect enhancer, L-Arg, together with Smaplatin^®^, with 50 mg/mouse L-Arg being injected ip. 

Tumor volumes and body weights were determined throughout the experimental period. After we measured the length (L) and width (W) of the tumors, we calculated tumor volume (mm^3^) as (W^2^ × L)/2.

### 2.7. Cytotoxicity of ISDN and Sildenafil Citrate in HeLa and C26 Cells 

In vitro cytotoxicity of ISDN and sildenafil was determined by using the MTT method with HeLa and colon carcinoma C26 cells. Both types of cells (1 × 10^4^ cells/well) were plated in 96-well culture plates and cultured overnight in D-MEM with 10% FBS and antibiotics (100U penicillin/mL and 100 µg/mL of streptomycin) at 37 °C under 5% CO_2_ and 95% air atmosphere. The medium was then replaced with fresh medium, and treatment proceeded with various concentrations of ISDN and sildenafil. After treatment, cells were incubated at 37 °C for 24 h. The MTT assay was then performed, and the toxicity was quantified as the fraction of surviving cells compared with that without drug treatment (control).

### 2.8. Statistical Analyses

In all experiments, error bars represent the standard deviation (SD) unless otherwise noted. Data were analyzed by using analysis of variance followed by the Bonferroni *t*-test. A difference was considered to be statistically significant when *p* < 0.05; *n* ≥ 5 samples for each group unless noted.

## 3. Results

### 3.1. Augmentation of Delivery of Nanomedicines to C26 Tumors in Mice by Using ISDN or Sildenafil Citrate

**Figure 1:**
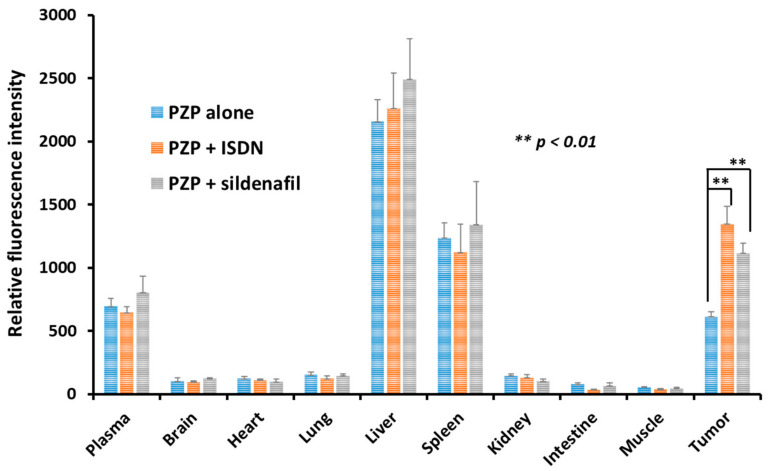
Enhancement of delivery of PZP to tumors by using EPR-effect enhancers. Male, 6-week-old, BALB/c mice bearing Colon 26 tumor were given 10 mg/kg PZP iv; ISDN at 30 mg/kg intraperitoneally or sildenafil citrate at 30 mg/kg subcutaneously immediately after PZP. The amount of drug in each tissue was quantified by using fluorescence spectroscopy, with the excitation wavelength of 422 nm (corresponding to ZnPP). Data are expressed as means ± SD. See text for details.

We investigated the use of ISDN and sildenafil citrate to increase delivery of different nanomedicines to advanced tumors that were 15–18 mm in diameter (about 2000–3000 mm^3^), in C26 tumors-model mice. The three nanomedicines used were PZP, SGB-complex, and Smaplatin^®^. Table S1 summarizes their characteristics [36,37,38]. We first determined tumor delivery of PZP by means of fluorescence spectroscopy. Data showed high accumulation of PZP in tumors except in the liver and spleen. When PZP was used in combination with ISDN or sildenafil citrate, tumor accumulation increased about two-fold at 24 h after iv administration of PZP compared with use of PZP alone but no EPR enhancers (Figure 1). As an interesting finding, drug delivery increased significantly only in tumor tissue; in other normal tissues, no significant drug accumulation was seen (Figure 1). Therefore, restoration of blood flow by using EPR-effect enhancers improved EPR effect-based drug delivery to this tumor. This finding (Figure 1) is consistent with our previous data: when P-HPMA-conjugated pirarubicin (P-THP) [39] or P-HPMA-conjugated pyropheophorbide [40] was administered in combination with an NO donor, NG, L-Arg, or HU, drug accumulation in tumors increased two- to three-fold in S180 and C26 tumor-bearing mice [12,25]. As a notable result, ISDN enhanced drug accumulation about 20% more than did sildenafil citrate (Figure 1). The reason for this result with ISDN is not clear, but one possibility may be that direct NO production by ISDN occurred selectively in tumor tissue.

**Figure 2:**
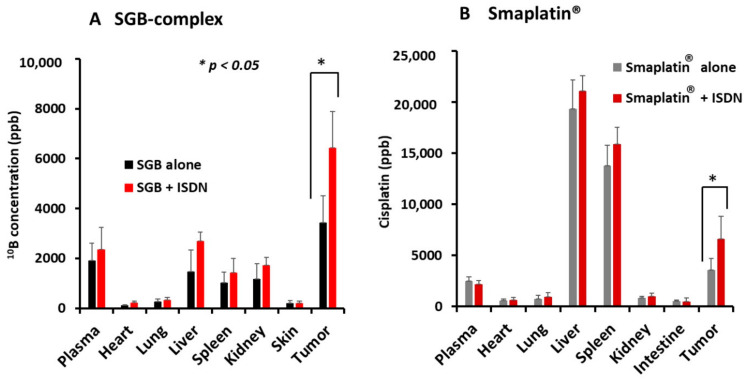
ISDN-enhanced accumulation of SGB-complex (**A**) and Smaplatin^®^ (**B**) in C26 tumor tissues. SGB-complex at 15 mg/kg (boric acid equivalent) or Smaplatin at 10 mg/kg (Cisplatin equivalent) was administered iv; 30 mg/kg ISDN was administered ip as an EPR-effect enhancer. At 24 h after drug treatment, the amounts of ^10^B and platinum in tissues were quantified by means of ICP-MS according to the manufacturer’s procedure. Data are expressed as means ± SD. (*n = 5*) See text for details.

We also studied delivery of SGB-complex and Smaplatin^®^ given with ISDN in the same tumor model and observed increased delivery of the boron in the SGB-complex and the platinum in Smaplatin^®^ to the tumor tissues, as determined by ICP-MS. We found that 15 mg/kg SGB-complex given iv in combination with 30 mg/kg ISDN given ip led to significantly enhanced delivery of ^10^B to tumor tissues by about two-fold at 24 h after drug administration; no other tissue demonstrated similar results (Figure 2A). Also, 10 mg/kg Smaplatin^®^ given with ISDN demonstrated increased accumulation in tumor tissues 1.5- to 2-fold at 24 h after iv infusion (Figure 2B). Again, these data indicate the importance of EPR-effect enhancers to increase delivery of drugs to late-stage tumors.

### 3.2. Enhanced Drug Delivery to Advanced Tumors by Using ISDN or Sildenafil Citrate as Revealed by Ex Vivo Fluorescence Imaging

**Figure 3:**
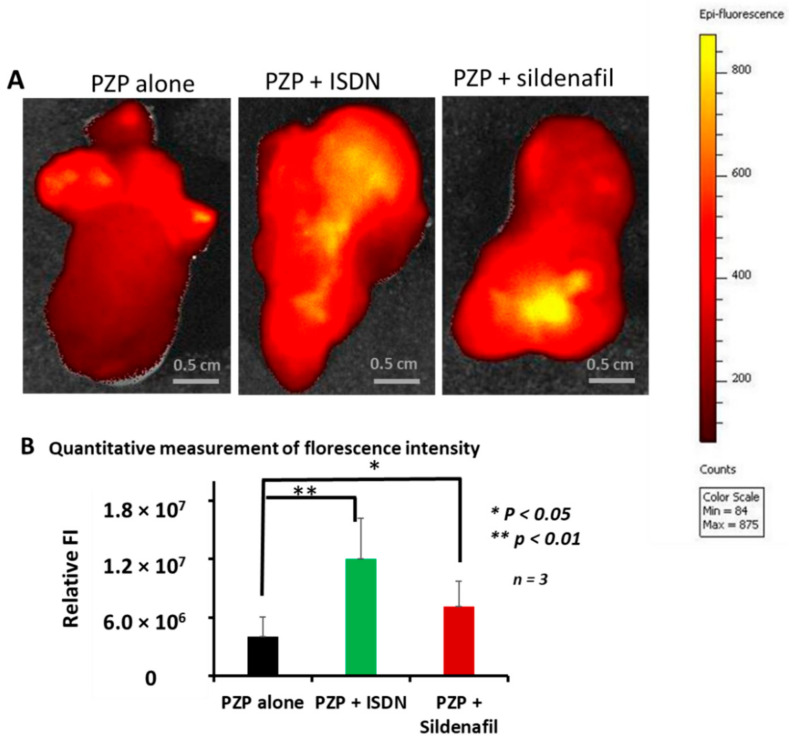
Ex vivo imaging of advanced mouse tumors after treatment with PZP plus ISDN or sildenafil. To study EPR-effect enhancers, we used late-stage C26 tumors (about 18 mm in diameter). PZP, 5 mg/kg, was injected iv, after which ISDN or sildenafil was administered. After 24 h of iv infusion, tumors were removed from mice, and fluorescence images were obtained by IVIS. Both enhancers augmented drug delivery to tumors about two- to three-fold (**A**). (**B**) shows the comparison quantitative measurement of PZP drug accumulation with/without EPR-effect enhancers based on the fluorescence intensity. Data are expressed as means ± SD (*n* = 3).

We continued to investigate the enhancement of delivery of PZP nanoparticles given alone or with ISDN or sildenafil citrate by using ex vivo fluorescence imaging by IVIS to study cut surfaces of tumor tissues. In this study, we utilized large-size tumors about 18 mm in diameter (3000 mm^3^), i.e., advanced C26 tumors in which tumor blood flow may be suppressed or blood vessels may be embolized by clots. Fluorescence intensity data showed that, again, ISDN enhanced drug delivery about three-fold at 24 h after PZP infusion compared with PZP alone (Figure 3A,B). Sildenafil citrate also improved PZP delivery about two-fold compared with PZP alone (Figure 3A,B). In addition, by using this ex vivo tumor-imaging method, we confirmed enhanced drug delivery with L-Arg, 50 mg/mouse: PZP accumulation increased about three- to four-fold after 24 h of iv infusion compared with PZP without L-Arg (Supplementary Figure S1).

### 3.3. Improvement in the Antitumor Effects of Nanomedicines by Using EPR-Effect Enhancers

**Figure 4:**
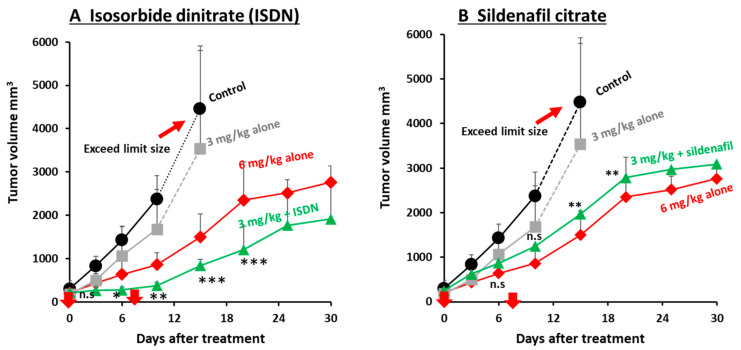
Improvement in the therapeutic effect of different concentrations of Smaplatin^®^ by using ISDN (**A**) and sildenafil (**B**) in C26 tumors. When tumor diameters measured about 12 mm, 6 mg/kg or 3 mg/kg Smaplatin^®^ was injected iv; ISDN (30 mg/kg, ip) or sildenafil (30 mg/kg sc) was given with 3 mg/kg Smaplatin^®^. The 3 mg/kg Smaplatin^®^ given with ISDN showed improved therapeutic efficacy compared with 6 mg/kg Smaplatin^®^ given alone (**A**); the result for 3 mg/kg Smaplatin^®^ plus sildenafil was similar to that for 6 mg/kg Smaplatin^®^ given alone (**B**). Arrows indicate times of drug administration. * *p* < 0.05, ** *p* < 0.01, *** *p* < 0.001, combination group vs Smaplatin^®^ 3 mg/kg group. Data are expressed as means ± SD. (*n = 5*).

We studied two EPR-effect enhancers—ISDN and sildenafil citrate—given in combination with different concentrations of the two micellar nanomedicines, Smaplatin^®^ and SGB-complex (Table S1), in C26 tumors. We found that 3 mg/kg Smaplatin^®^ given with 30 mg/kg ip ISDN resulted in a better therapeutic effect at day 30 than that for 6 mg/kg Smaplatin^®^ given alone (no ISDN) (Figure 4A). In contrast, 3 mg/kg Smaplatin^®^ given alone showed very little antitumor effect at day 12 or later (Figure 4A). These data suggest that ISDN enhanced the therapeutic effect of Smaplatin^®^ about three- to four-fold in C26 tumor-bearing mice, a result that was consistent with our previous findings for P-THP given in combination with NO-generating agents (NG, L-Arg, and HU) [7,10,25], all of which selectively generated NO in tumor tissues. These agents produced two- to three-fold greater antitumor effects in various tumor models [7,10,25]. 

Smaplatin^®^, 3 mg/kg, given with 10 mg/kg sildenafil citrate sc suppressed tumor growth about 1.5-fold at day 7 after treatment (Figure 4B). To study whether the therapeutic effect would be enhanced by using a different sildenafil citrate concentration, we used 30 mg/kg sildenafil citrate at day 8 and found that the combination treatment enhanced the antitumor effect about two-fold at day 30 (Figure 4B).

We also found that the combination treatment of Smaplatin^®^ with L-Arg in the C26 tumor model improved the therapeutic efficacy of Smaplatin^®^ two-fold (Supplementary Figure S2).

**Figure 5:**
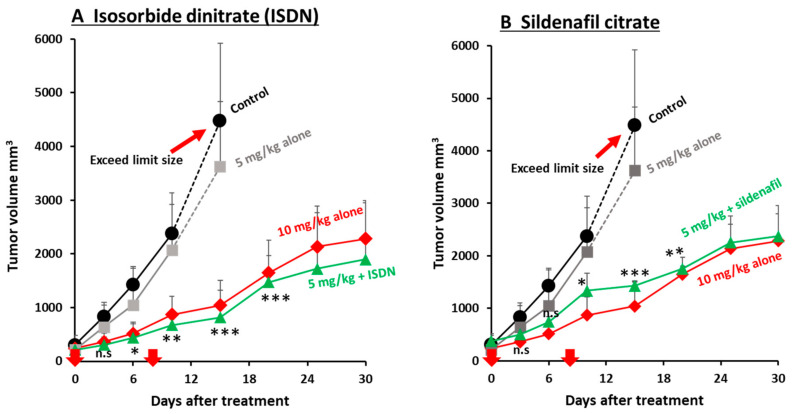
Improvement in the antitumor effect of SGB-complex by using EPR-effect enhancers in the C26 tumor model. (**A**) Antitumor effect of the SGB-complex given with ISDN. (**B**) Antitumor efficacy of the SGB-complex given with sildenafil. ISDN increased the antitumor effect of the SGB-complex about 3-fold; sildenafil, 1.5- to 2-fold. Arrows indicate times of drug administration. Data are expressed as means ± SD. * *p* < 0.05, ** *p* <0.01, *** *p* < 0.001, combination group vs. SGB 5 mg/kg group. See text for details.

In addition, we confirmed an enhanced therapeutic effect by using another micellar drug, SGB-complex, which was developed for BNCT. We previously reported that SGB-complex itself suppressed tumor growth in vivo and in vitro by inhibiting glycolysis and by damaging functions of mitochondrial membranes in cancer cells [37]. To increase the antitumor effect of the SGB-complex, in our study here, we used ISDN or sildenafil citrate as an EPR-effect enhancer. We observed almost no therapeutic effect when 5 mg/kg SGB-complex alone was infused iv, whereas 5 mg/kg SGB-complex given iv in combination with 30 mg/kg ISDN produced an enhanced antitumor effect compared with that for 10 mg/kg SGB-complex given alone (Figure 5A). These data suggest that combination therapy with ISDN can enhance the therapeutic effect of nanomedicines about two- to three-fold. Sildenafil citrate was also given twice, at day zero (10 mg/kg sc) and day eight (30 mg/kg sc), and we found an improved antitumor effect of about two-fold (Figure 5B). 

To see the cytotoxicity of ISDN and sildenafil, we examined two cancer cells, e.g., HeLa and C26, and we found both EPR-effect enhancers did not show any significant cytotoxicity in both cells after 24 h incubation based on MTT assay (Supplementary Figure S3). Furthermore, we evaluated the in vivo toxicity of Smaplatin^®^ (Supplementary Figure S4A) and SGB-complex (Supplementary Figure S4B) with or without EPR-effect enhancer (ISDN or sildenafil) by monitoring the mouse body-weight changes. We observed that the combination treatment did not exhibit any notable body-weight changes up to 30 days after treatment (Supplementary Figure S4).

## 4. Discussion

Commonly used cancer chemotherapeutic agents, including immunotherapy drugs, have shown failure rates of 90% (±5%) for solid tumors [15]. One reason for this low success rate is that most drugs currently used in clinics to treat solid tumors are low-molecular-weight compounds. As a consequence, these drugs travel throughout the body and diffuse indiscriminately when administered iv. They thus cause severe adverse effects in normal tissues and organs and result in a lower therapeutic effect [7,9,12,15,24,41,42]. In contrast, when biocompatible nanodrugs are administered iv, they remain in circulating blood for a long time, and they gradually penetrate tumor tissue and selectively accumulate there because of the EPR effect [7,15,24]. One criticism raised about the EPR effect was that this effect was not observed in human cancers. However, Lee et al. recently demonstrated the presence of the EPR effect in breast cancer, including metastatic tumors [20], and Ding et al. reported a positive EPR effect in about 87% of patients with renal cancer [19]. These data are consistent with the clinical findings of Maeda’s group with SMANCS/lipiodol infused into tumor-feeding arteries, with this method showing remarkable results. Their method of using an arterial infusion of SMANCS/lipiodol (drug) selectively delivered the drug to tumors by virtue of the EPR effect, and the selective delivery was clearly visualized by using CT [3,5,11,12,22,23,24]. Also demonstrated in radio scintigraphy imaging of a tumor using γ-emitting gallium-67 citrate, when it was infused iv, it formed a complex with transferrin (90 kDa) in the plasma. This complex behaves as a nanomedicine. After 48–72 h, this complex accumulated selectively in solid tumors, as visualized by a γ-scintillation camera [43], which provides clear evidence of the EPR effect.

The heterogeneity of the EPR effect presents another problem in that tumor blood vessels are frequently embolized or occluded, and blood flow is suppressed, as discussed above. When tumor blood flow is obstructed, no typical EPR effect is observed even when nanomedicines are administered [7,8,18,24]. We had previously reported restoration of obstructed tumor blood flow by using NO donors, L-Arg, NG, and HU [7,10,11,12,25], and micellar forms of CO donors such as SMA/CORM2 and PEG-hemin or an inducer of heme oxygenase-1 (HO-1), which also generates CO [26,27]. These vascular mediators remarkably increased the tumor delivery of nanomedicine by augmenting tumor blood flow and vascular permeability, consequently improving the therapeutic efficacy of nanomedicines two- to three-fold in various tumor models in mice and rats and also in humans [23,44].

In this report, we confirmed that the two EPR-effect enhancers ISDN and sildenafil citrate, in addition to L-Arg, improved delivery of three different micellar drugs—Smaplatin^®^, SGB-complex, and PZP—to tumors, and we also confirmed the therapeutic effect of these three nanomedicines on C26 tumors in mice. Table S1 summarizes the physicochemical properties of these three nanomedicines. 

We first investigated the delivery of PZP to mouse tumors by using ISDN and sildenafil citrate, which enhanced delivery about two-fold (Figure 1). We previously showed that PZP produced an excellent anticancer effect in various tumor models when used with light irradiation in PDT. In addition, even without light irradiation, PZP also suppressed HO-1 in cancer cells [38,45], inhibited HSP-32 (tumor survival factor), and downregulated oncogene expression so that it ultimately suppressed tumor growth [46,47]. PZP showed relative high liver and spleen accumulation, which is commonly seen for many nanomedicines because liver and spleen are rich in reticuloendothelial systems to capture macromolecules. However, NO donors did not significantly increase the drug accumulation in liver and spleen but only remarkably increased tumor accumulation, suggesting these EPR enhancers will not increase the side effects of nanomedicine. Furthermore, for PZP that is used for PDT upon light irradiation, the side effects or toxicities to the liver and spleen are not significant because light irradiation is applied to the tumor but not to the liver or spleen. Smaplatin^®^ is a pH-sensitive micellar drug (cisplatin complexed with SMA polymer [36]) that releases free cisplatin in an acidic milieu after tumor-selective accumulation, and the released cisplatin inhibits DNA synthesis of cancer cells [36]. We showed that ISDN improved delivery of these nanomedicines to tumors, thereby increasing anticancer efficacy more than 3-fold; sildenafil improved delivery 1.5- to 2-fold (Figure 2B and Figure 4A). We also confirmed that the use of SGB-complex with ISDN resulted in an improved therapeutic effect, by two- to three-fold, in the same tumor model and by the same mechanisms (Figure 2A and Figure 5A). 

In Figure 1 and Figure 2, high uptake is seen for PZP and Smaplatin^®^ in the liver and spleen, so called by reticuloendothelial system (RES). To suppress this uptake, we are now investigating how to avoid this issue by pretreating lipid microparticles of Intralipid^®^ or lipiodol by blocking scavenger receptor of RES. This strategy seems effective to prevent RES uptake [48]

SGB-complex was initially designed for use in BNCT. We discovered by chance that this complex can inhibit hypoxia-adapted tumor-cell growth under mildly hypoxic conditions (pO_2_, 6–10%) by inhibiting glycolysis and by damaging functions of mitochondrial membranes in cancer cells [37] without neutron irradiation. SGB-complex also significantly suppressed tumor growth two- to three-fold when used with ISDN in vivo compared with SGB-complex alone (Figure 5A). We later confirmed a much improved therapeutic effect after utilization of neutron irradiation [37]. Therefore, we expect that the use of EPR-effect enhancers plus neutron irradiation will enhance the therapeutic effects of this method even more. Experiments investigating this possibility are under way and should usher in a new era in BNCT.

We also confirmed the EPR-enhancing effect of sildenafil citrate. The mode of action of sildenafil citrate is not by generation of NO, but the ultimate result of using it was similar to that related to NO generation. Das and Fisher and their colleagues showed that sildenafil citrate enhanced apoptosis and the antitumor efficacy of doxorubicin in a xenograft model of prostate-tumor-bearing mice [49,50]. This finding may be attributed to the EPR-enhancing effect. We report here that sildenafil citrate enhanced the delivery of PZP to advanced tumors by two- to three-fold, as judged by fluorescence imaging (Figure 1 and Figure 3); it also improved the anticancer efficacy of both SGB-complex and Smaplatin^®^ by about 1.5- to 2-fold (Figure 4B and Figure 5B). Moreover, we confirmed that ISDN or sildenafil citrate itself did not show any toxicity in vitro (Supplementary Figure S3), and in the combination treatment with nanomedicine, we did not find significant body-weight loss in mice (Supplementary Figure S4A,B). These data suggest that EPR enhancers play a critical role for the improvement of tumor drug delivery of nanomedicines and thus enhancement of the therapeutic effect.

## 5. Conclusions

We present here examples of the effective influence of the EPR enhancers ISDN and sildenafil citrate, which we evaluated with three micellar polymer drugs—SGB complex, Smaplatin^®^, and PZP—in a mouse model with relatively advanced C26 tumors. Smaplatin^®^ releases free cisplatin in tumor tissues and damages cancer cells by inhibiting their DNA synthesis [36]. SGB-complex, in contrast, generates free boric acid in tumors which exhibit microenvironmental acidic pH, and boric acid thus generated competes with phosphate in phosphorylation reaction of glucose by hexokinase in glycolysis. As a result, it inhibits glycolysis in cancer cells; the glucosamine moiety of this complex seems to damage functions of mitochondrial membranes in cancer cells [37]. We also showed that the delivery to tumors and antitumor effects of Smaplatin^®^ and SGB-complex were enhanced by using ISDN and sildenafil citrate by about 2- to 4-fold and 1.5- to 2-fold, respectively (Figure 2, Figure 4 and Figure 5). A similar EPR effect-enhancing result was observed with another fluorescent macromolecular drug PZP, with two-fold enhancement (Figure 1 and Figure 3). Previous studies of the NO donors L-Arg, NG, and HU and the ACE inhibitor (ACE-1) showed about two-fold enhancement of therapeutic effects in the same C26 and S180 tumors in mice. These data further confirm the efficacy of EPR-effect enhancers in the treatment of advanced cancer.

## Data Availability

The data presented in this study are available on request from the corresponding author or first author of this article.

## Supplementary Materials

The following are available online at https://www.mdpi.com/article/10.3390/jpm11060487/s1, Figure S1: Enhancement of tumor drug delivery of PZP using L-arginine evaluated by fluorescence imaging (IVIS), Figure S2: Improvement of therapeutic effect of Smaplatin^®^ by using L-arginine in C26 tumor, Figure S3: Cytotoxicity of ISDN and sildenafil citrate in HeLa and C26 cells, Figure S4: In vivo toxicity of Smaplatin^®^ and SGB-complex with EPR effect enhancers revealed by body weight change, Table S1: Characteristics of micellar drugs used in this study.
