# Supplementary material for: EPR-Effect Enhancers Strongly Potentiate Tumor-Targeted Delivery of Nanomedicines to Advanced Cancers: Further Extension to Enhancement of the Therapeutic Effect
Source: J Pers Med. 2021 May 28;11(6):487. doi: 10.3390/jpm11060487 (PMC8229906; doi:10.3390/jpm11060487)
Supplement: Supplementary file 1 [file jpm-11-00487-s001.zip › jpm-1208499-supplementary.pdf]

Journal of Personalized Medicine
MDPI

Supplementary data

EPR Effect Enhancers Strongly Potentiate Tumor-Targeted Delivery of Nanomedicines to Advanced Cancers: Further extension to Enhancement of the Therapeutic Effect

Waliul Islam[1,5], Shintaro Kimura[1,2], Rayhanul Islam[3], Ayaka Harada[4], Katsuhiko Ono[1], Jun Fang[3], Takuro Niidome[4], Tomohiro Sawa[1], Hiroshi Maeda[1,5,6 *]

*Corresponding author. Email: maedabdr@sweet.ocn.ne.jp

The supplementary data include:

Table S1. Characteristics of micellar drugs used in this study

Figure S1. Enhancement of tumor drug delivery of PZP using L-arginine evaluated by fluorescence imaging (IVIS).

Figure S2. Improvement of therapeutic effect of Smaplatin® by using L-arginine in C26 tumor.

Figure S3. Cytotoxicity of ISDN and sildenafil citrate in HeLa and C26 cells.

Figure S4. In vivo toxicity of Smaplatin® and SGB-complex with EPR effect enhancers revealed by body weight change.

Table S1. Characteristics of micellar drugs used in this study.

Property	PZP[a]	Smaplatin®[a]	SGB-complex[a]
i) Hydrodynamic diameter obtained by DLS (nm)	82.8	102.5	12-15
ii) Surface charge (mV)	+ 1.12	- 20.9	- 37
iii) API content (wt%)	20	20.1	7-8
iv) Enhancement of tumor delivery	2-fold	1.5- to 2-fold	2-fold
v) Augmentation of therapeutic effect	2- to 3-fold	2- to 4-fold	2- to 3-fold

[a]PZP, HPMA polymer-conjugated zinc protoporphyrin [38]; Smaplatin®, complex of poly(styrene-co-maleic acid) (SMA) and cisplatin [36]; SGB-complex, SMA glucosamine-conjugated boric acid complex [37]; API, active pharmaceutical ingredient; DLS, dynamic light scattering. See text for details.

Figures

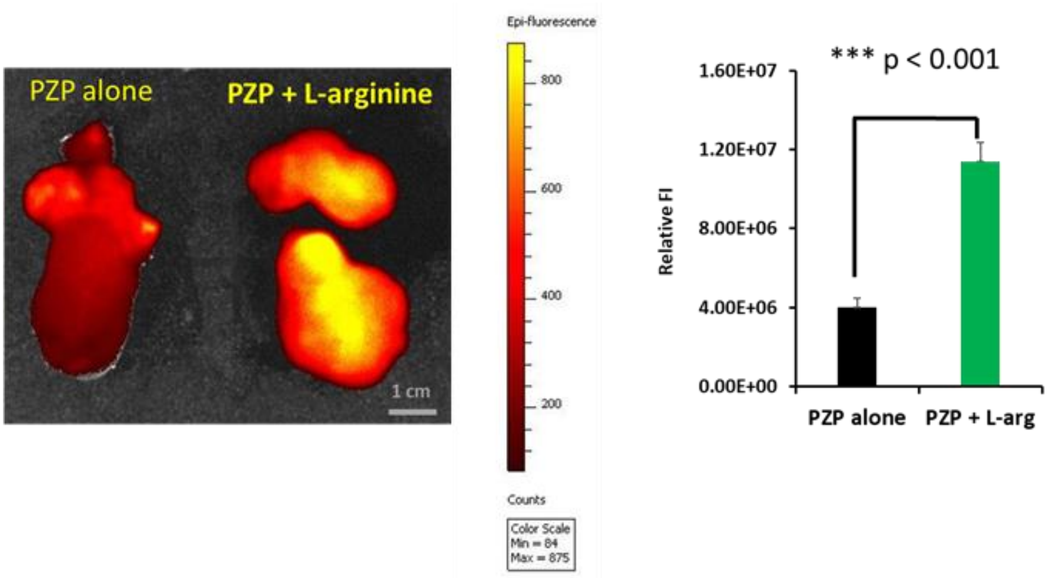

Figure S1. **Enhancement of tumor drug delivery of PZP using L-arginine evaluated by fluorescence imaging**. When tumor diameter reached about 15-18 mm then PZP at dose 5 mg/kg was infused iv and L-arginine at dose 50 mg/mouse was applied ip. After 24h of iv injection the ex vivo fluorescence image was conducted by IVIS system. Data are expressed as means ± SD. See text for detail.

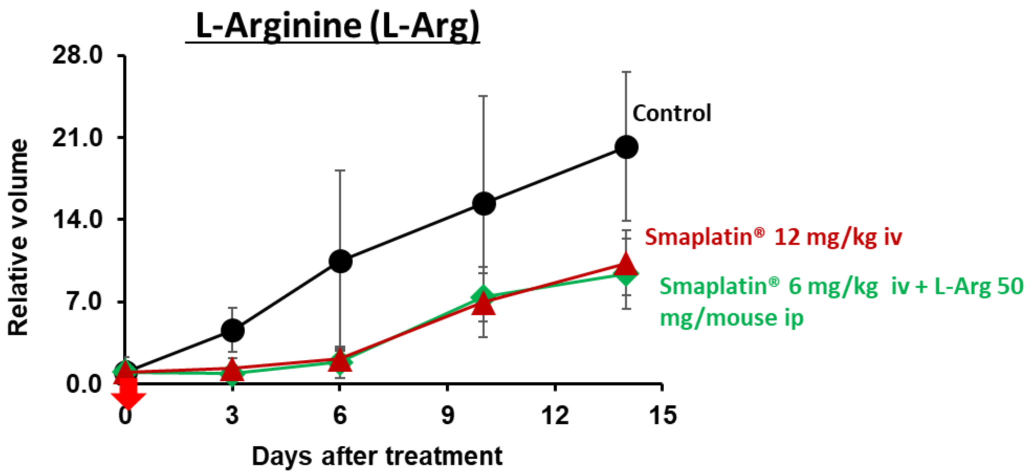

Figure S2. Improvement of therapeutic effect of Smaplatin® by using L-arginine in C26 tumor. When tumor size was about 12-14 mm then Smaplatin® at dose 12 mg/kg and 6 mg/kg were injected iv and L-arginine was applied as described above. Data are expressed as means ± SD. See text for detail.

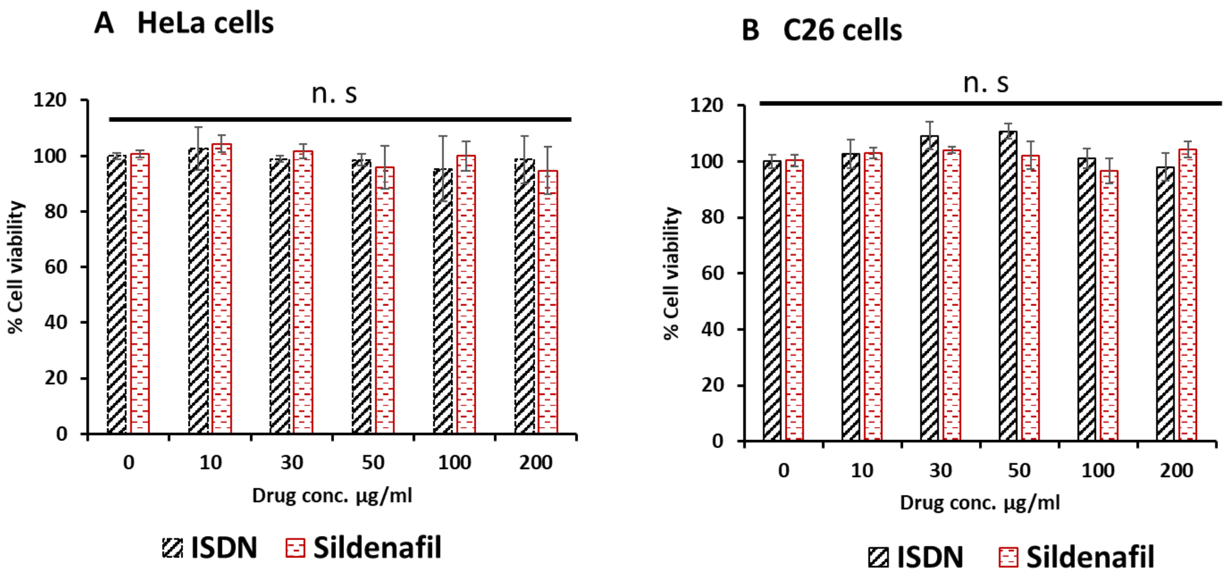

Figure S3. Cytotoxicity of ISDN and sildenafil citrate in HeLa (A) and C26 cells (B). HeLa and colon carcinoma C26 cells (1×10^4 cells/well) were plated in 96-well culture plates and cultured overnight in D-MEM medium. The medium was then replaced with fresh medium, and treatment proceeded with various concentrations of ISDN and sildenafil. After treatment, cells were incubated at 37ºC for 24 h and then MTT assay was performed. Data are expressed as means ± SD. See main text for detail.

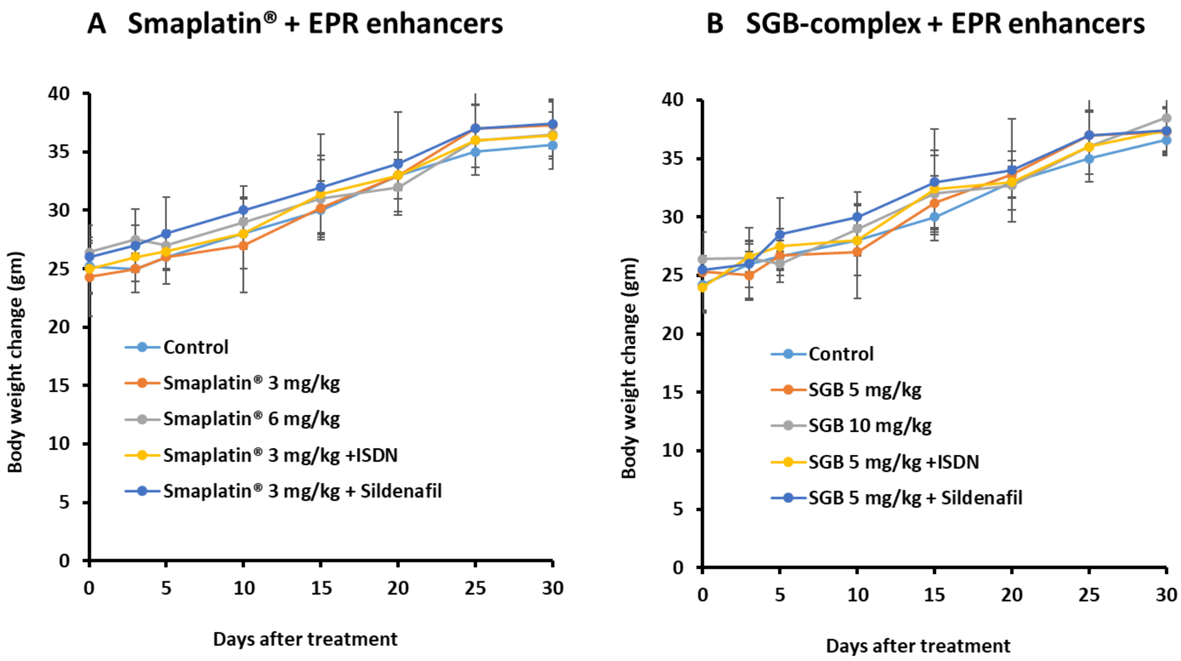

Figure S4. In vivo toxicity of Smaplatin® and SGB-complex revealed by body weight changes. BALB/C 6 weeks old mice were used in this study. When tumor diameter was about 12-14 mm then Smaplatin® and SGB-complex were injected iv. EPR effect enhancers ISDN 30 mg/kg ip and sildenafil 30 mg/kg sc were applied immediately after drug infusion. The body weight changes were monitored throughout the experiment. Data are expressed as means ±SD (n = 5)
